# Feedback regulation of small RNA processing by the cleavage product

**DOI:** 10.1080/15476286.2019.1612693

**Published:** 2019-05-22

**Authors:** Svetlana Durica-Mitic, Boris Görke

**Affiliations:** Department of Microbiology, Immunobiology and Genetics, Max F. Perutz Laboratories (MFPL), University of Vienna, Vienna Biocenter (VBC), Vienna, Austria

**Keywords:** Small RNA GlmZ, RNase E, adaptor protein RapZ, RNA processing, feedback regulation

## Abstract

Many bacterial small RNAs (sRNAs) are processed resulting in variants with roles potentially distinct from the primary sRNAs. In *Enterobacteriaceae* sRNA GlmZ activates expression of *glmS* by base-pairing when the levels of glucosamine-6-phosphate (GlcN6P) are low. GlmS synthesizes GlcN6P, which is required for cell envelope biosynthesis. When dispensable, GlmZ is cleaved by RNase E in the base-pairing sequence. Processing requires protein RapZ, which binds GlmZ and recruits RNase E by interaction. Cleavage is counteracted by the homologous sRNA GlmY, which accumulates upon GlcN6P scarcity and sequesters RapZ. Here, we report a novel role for a processed sRNA. We observed that processing of GlmZ is never complete *in vivo*. Even upon RapZ overproduction, a fraction of GlmZ remains full-length, while the 5ʹ cleavage product (GlmZ*) accumulates. GlmZ* retains all elements required for RapZ binding. Accordingly, GlmZ* can displace full-length GlmZ from RapZ and counteract processing *in vitro*. To mimic GlmZ* *in vivo*, sRNA chimeras were employed consisting of foreign 3ʹ ends including a terminator fused to the 3ʹ end of GlmZ*. *In vitro*, these chimeras perform indistinguishable from GlmZ*. Expression of the chimeras *in vivo* inhibited processing of endogenous GlmZ, causing moderate upregulation of GlmS synthesis. Hence, accumulation of GlmZ* prevents complete GlmZ turnover. This mechanism may serve to adjust a robust *glmS* basal expression level that is buffered against fluctuations in RapZ availability.

## Introduction

Bacterial *trans*-encoded small RNAs (sRNAs) cooperate extensively with ribonucleases (RNases) to exert their regulatory roles [1,2]. In Gram-negative bacteria, the endoribonuclease RNase E holds a key role, not only for bulk RNA turnover, but also for sRNA function [3,4]. Many sRNAs guide RNase E to destabilize their target RNAs [5–7], which often involves co-degradation of the base-paired sRNA [8–10]. Often, RNase E is also responsible for initiation of degradation of *trans*-encoded sRNAs when not base-paired [2]. However, RNase E also has important roles for sRNA biogenesis and maturation. Several sRNAs are known to be produced from the 3ʹ region of protein coding genes, either through usage of an internal promoter or by RNase E catalyzed mRNA processing [11–13], and regulatory roles for sRNAs generated through the latter mechanism were demonstrated in various species including *E. coli* and *Salmonella* [14–17]. An additional class of sRNAs is autonomously transcribed, but undergoes maturation. RNase E converts these sRNAs into shorter stable variants that retain regulatory potential [18–21]. At least in one case, sRNA maturation was shown to be essential for regulation [12]. In addition, processing by RNase E may generate sRNA species with a target spectrum distinct from the genes regulated by the unprocessed sRNA [22]. sRNAs processed in the 5ʹ region carry a 5ʹ monophosphate converting them into preferred substrates for further degradation by RNase E. The sRNA 5ʹ monophosphate may also promote rapid target RNA degradation through interaction with the 5ʹ sensing pocket in RNase E, thereby allosterically activating the enzyme [23]. Taken together, processed variants of sRNAs may have specific roles and properties deviating from the corresponding full-length sRNAs.

How RNase E selects its cleavage sites is weakly understood. A study mapping RNase E cleavage sites genome-wide proposed the degenerate cleavage motif RN|WUU [12], suggesting that additional elements such as RNA secondary structures contribute to RNase E recognition [24]. Indeed, in-depth analysis of sRNA MicL revealed that precise cleavage by RNase E is directed by two adjacent stem loop structures located 3ʹ to the cleavage site [25]. Furthermore, dedicated adaptor proteins may be employed to mediate precise cleavage of sRNAs in a regulated manner. A prominent example is provided by the GlmY/GlmZ/RapZ circuit in *Enterobacteriaceae*, which controls synthesis of enzyme GlmS at the post-transcriptional level [26]. GlmS produces glucosamine-6-phosphate (GlcN6P), which is required for synthesis of cell envelope components peptidoglycan and LPS. When GlcN6P levels are low, the small RNA GlmZ base-pairs with the *glmS* 5ʹ-UTR to activate translation, which concomitantly stabilizes the mRNA [27,28]. GlmZ is constitutively transcribed and regulated at the level of decay. When not required, the adaptor protein RapZ binds GlmZ and recruits RNase E through interaction to inactivate the sRNA by cleavage in the base-pairing region [29,30]. GlmZ consists of three stem loop structures, the central of which is decisive for RapZ binding and cleavage by RNase E that occurs 6 or 7 nt downstream of this structure [31]. Due to coinciding sites, binding of Hfq and cleavage by the RapZ/RNase E complex are mutually exclusive. Cleavage of GlmZ is regulated through employment of the homologous sRNA GlmY, which accumulates when the GlcN6P concentration decreases [32]. GlmY serves as decoy and sequesters RapZ thereby counteracting GlmZ processing, but is itself not cleaved by RNase E [30,31].

In the current study, we addressed whether the processed variant of GlmZ (subsequently designated GlmZ*) also has a role. We observed that processing of GlmZ is never complete *in vivo* suggesting that accumulation of GlmZ* limits ongoing GlmZ cleavage. Indeed, GlmZ* efficiently competes with full-length GlmZ for binding to RapZ. Accordingly, GlmZ* is capable of inhibiting cleavage of full-length GlmZ by RapZ/RNase E *in vitro*. Finally, expression of sRNA chimeras mimicking GlmZ* counteracted processing of endogenous GlmZ *in vivo* and caused a moderate upregulation of *glmS* expression, which was more pronounced in strains lacking decoy sRNA GlmY. We conclude, that the level of GlmZ* sets a threshold to prevent complete turnover of full-length GlmZ. This mechanism may serve to provide a robust basal level of *glmS* expression, which might be in particular important when the level of the decoy sRNA GlmY is low.

## Results and discussion

### Even extraordinary high Rapz levels do not trigger complete processing of GlmZ *in vivo*

**Figure 1.**
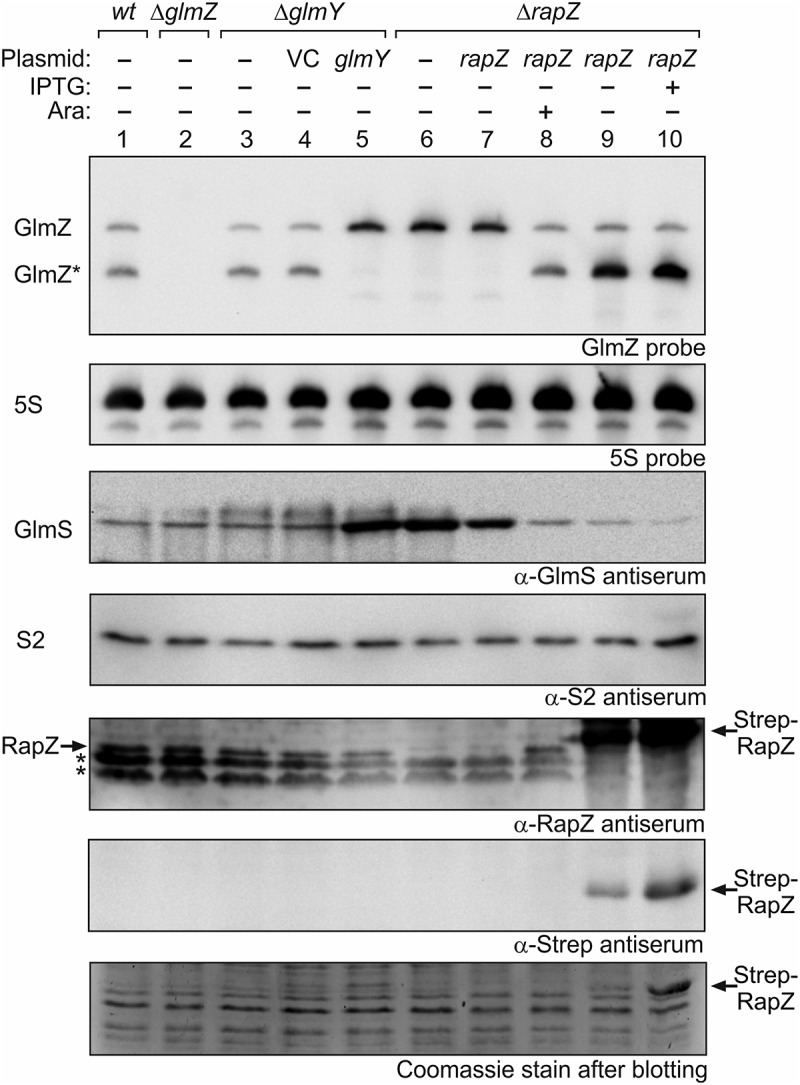
High RapZ concentrations trigger accumulation of processed GlmZ but do not completely turn over full-length GlmZ. Northern analyses addressing abundance of full-length and processed GlmZ in various genetic backgrounds (top panel) and the consequences for GlmS protein levels as detected by immunoblot analyses (panel 3 from top). Strains R1279 (*wild type*, lane 1), Z45 (*ΔglmZ*, lane 2), Z95 (*ΔglmY*, lanes 3–5) and Z37 (*ΔrapZ*, lanes 6–10) were used, which occasionally harboured the following plasmids: pBR-plac (VC = vector control; lane 4), pYG83 (lane 5), pBGG61 (lanes 7, 8), pBGG164 (lanes 9, 10). The bacteria were grown to exponential phase and harvested for isolation of total RNA and total protein, respectively. 5 μg of the total RNA preparations were subjected to Northern analysis using probes directed against GlmZ (top panel) and 5S rRNA as loading control (second panel from top). Total protein extracts were separated by SDS PAGE and analysed by Western blotting using antisera directed against GlmS (panel 3 from top), ribosomal protein S2 as loading control (panel 4 from top), RapZ (panel 5 from top), and the Strep epitope (panel 6 from top). Non-specific signals are indicated with asterisks. Following blotting, the SDS PAA gel was stained with Coomassie blue to visualize overproduced Strep-RapZ and to provide a further loading control (bottom panel). Arabinose and IPTG were added to the cultures for induction of *rapZ* expression as indicated.

In *wild type* cells grown to exponential phase, slightly more GlmZ* than full-length GlmZ is detectable in Northern analysis (Figure 1, lane 1 [30]). A *glmY* deletion has only a limited impact and shifts this ratio slightly in favour of GlmZ* as observed previously (Figure 1, lanes 3 and 4 [30,32];. Physiologically, GlmY accumulates and strongly counteracts GlmZ processing when cells experience GlcN6P scarcity [32,33]. Plasmid-driven overexpression of GlmY abolishes GlmZ processing and leads to strong upregulation of GlmS (Figure 1, lane 5), which is the consequence of sequestration of RapZ by GlmY [30]. A comparable result was obtained in the *ΔrapZ* mutant, reflecting that RapZ is absolutely required to guide GlmZ to processing by RNase E (Figure 1, lane 6 [30];). Complementation of the *ΔrapZ* mutant with a low copy plasmid carrying *rapZ* under control of the tightly regulated *P_BAD_* promoter restores processing of GlmZ in the presence of the inducer arabinose (Figure 1, lanes 7–8). We performed a similar complementation experiment but used a plasmid with the IPTG-inducible *P_tac_* promoter and a strong ribosomal binding site to overproduce RapZ to higher levels. This construct allows overproduction of RapZ, which carried an N-terminal Strep-tag in this case, to levels becoming visible even in total cell extracts analysed by SDS-PAGE/Coomassie staining. Strep-RapZ was already detectable in the absence of IPTG (due to leakiness of *P_tac_* repression by LacI) and accumulated further in the presence of IPTG (Figure 1, bottom panel, lanes 9–10). Western blotting using antisera directed against RapZ or the Strep-tag validated this band as RapZ (Figure 1, panels 5 and 6, lanes 9–10). Interestingly, regardless of these high RapZ levels, complete processing of GlmZ was not obtained (Figure 1, top panel, lanes 9–10). GlmZ* accumulated to high levels, whereas the level of full-length GlmZ remained basically unaffected (Figure 1, top panel, compare lanes 9–10 with lanes 1 and 8). Thus, the extent of GlmZ processing does not strictly correlate with the RapZ level and is limited by another factor. As it strongly accumulates upon RapZ overproduction, we reasoned that this factor might be GlmZ*.

### GlmZ* counteracts cleavage of full-length GlmZ by inhibition of RapZ/GlmZ complex formation *in vitro*

**Figure 2.**
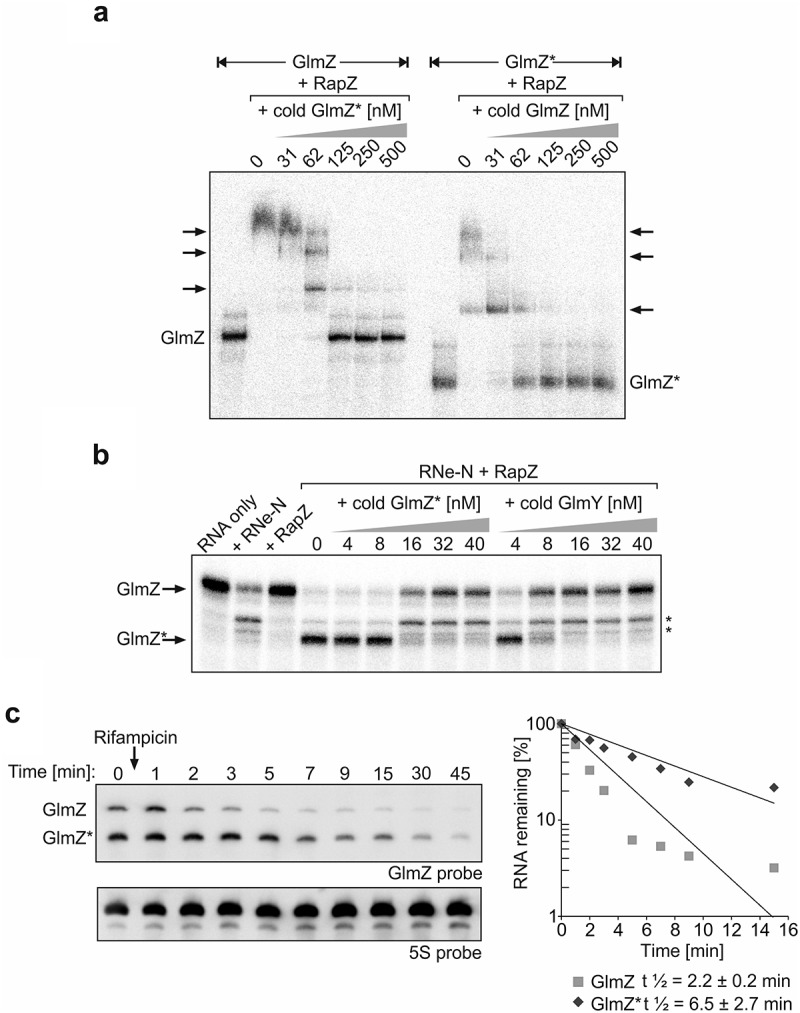
GlmZ* competes efficiently with full-length GlmZ for binding RapZ and inhibits cleavage of full-length GlmZ by RNase E *in vitro*. (a) EMSA demonstrating that full-length and processed GlmZ can displace each other from complexes with RapZ. α-^32^P-UTP labelled full-length GlmZ (left) and GlmZ* (right) were pre-incubated with 1500 nM RapZ to achieve complex formation. Subsequently, incremental concentrations of non-labelled GlmZ* (left) and full-length GlmZ (right) were added, respectively. Following an additional incubation step, binding reactions were separated by non-denaturing gel electrophoresis and gels were analysed by phospho-imaging. (b) *In vitro* cleavage assay demonstrating that GlmZ* inhibits cleavage of full-length GlmZ by RapZ/RNase E. α-^32^P-UTP labelled full-length GlmZ was incubated alone (‘RNA only’, lane 1) or solely with 25 nM RNase E-NTD (lane 2) or 20 nM RapZ (lane 3), or with both proteins simultaneously (lanes 4–14). In the latter case, various concentrations of non-labelled GlmZ* or GlmY were added as indicated. Following incubation, the reaction products were separated on denaturing PAA gels, which were subsequently analysed by phospho-imaging. Non-specific cleavage products are indicated by asterisks on the right. (c) Left: Northern blot addressing half-life of full-length and processed GlmZ following treatment with rifampicin. *Wild* *type* strain R1279 was grown to exponential phase and treated with rifampicin to stop transcription. At indicated times samples were removed for isolation of total RNA, which was analysed by Northern blotting using probes directed against GlmZ (top) and the 5S rRNA (bottom). Right: Semi-logarithmic plots of GlmZ* and full-length GlmZ decay for half-life determination. RNA signal intensities were normalized to 5S signals and plotted semi-logarithmically in percent against time. The graph shows the average values of three independent experiments.

So far, no condition has been identified that would enable complete processing of GlmZ *in vivo*. We previously showed that Hfq provides protection against cleavage by RapZ/RNase E by covering the RNase E cleavage site in GlmZ upon binding [31]. Accordingly, abundance of full-length GlmZ decreases in favour of GlmZ* in an *hfq* mutant. Nonetheless, a minor but significant fraction of GlmZ remains uncut even in the absence of Hfq [30]. The same is true for *ΔglmY* strains eliminating sequestration of RapZ by the decoy sRNA [32]. Taken together, GlmZ is not completely processed even when access of RapZ/RNase E to GlmZ is facilitated through elimination of Hfq or GlmY [30,32], or when RapZ is overproduced to high levels (Figure 1). The concomitant strong accumulation of GlmZ* in the latter case prompted us to inquire whether GlmZ* competes with full-length GlmZ for binding RapZ, thereby sequestering this protein and limiting cleavage of full-length GlmZ. Previous results have shown that RapZ requires the two 5ʹ-terminal stem loop structures in GlmZ for binding, which are also retained in GlmZ* [31]. Accordingly, full-length and processed GlmZ are both bound by RapZ with comparable affinities [29,30]. Therefore, we tested whether GlmZ* is able to displace full-length GlmZ from complexes with RapZ and vice versa, which we addressed by EMSA. In this case, radioactively labelled full-length GlmZ as well as GlmZ* were pre-incubated with saturating amounts of RapZ to allow for binding and subsequently increasing concentrations of ‘cold’ GlmZ* and full-length GlmZ were added, respectively. Indeed, GlmZ* displaced full-length GlmZ from the RapZ complexes with similar efficiency as observed in the reverse assay, which addressed dissolution of RapZ/GlmZ* complexes by cold full-length GlmZ (Figure 2(a)). In conclusion, processed and full-length GlmZ compete with comparable efficiencies for getting access to RapZ.

Next, we tested whether GlmZ* is capable of counteracting cleavage of full-length GlmZ by RNase E/RapZ *in vitro*, as previously observed for GlmY [30]. To this end, radioactively labelled full-length GlmZ was incubated with purified RapZ and the catalytic domain of RNase E in the absence or presence of incremental concentrations of unlabelled GlmZ* or GlmY for comparison to follow GlmZ cleavage within 30 min reaction time. RapZ has no role for GlmZ processing in the absence of RNase E, whereas incubation of GlmZ with RNase E alone yielded unspecific cleavage products only (Figure 2(b), lanes 1–3). In agreement with previous data [30,31], accumulation of properly processed GlmZ could only be observed in the presence of both, RapZ and RNase E-NTD (Figure 2(b), lane 4). Indeed, additional presence of GlmZ* in the assay inhibited GlmZ cleavage when supplied at concentrations of ≥16 nM (Figure 2(b), lanes 7–9). Inhibition of GlmZ cleavage by GlmZ* was less efficient as compared to GlmY, which impeded GlmZ processing already at a concentration of ≥8 nM (Figure 2(b), lanes 10–14). Taken together, GlmZ* competes with full-length GlmZ for RapZ binding, thereby counteracting GlmZ processing *in vitro*, but with a somewhat lower efficiency than observed for GlmY.

### Design of sRNA chimeras mimicking GlmZ*

**Figure 3.**
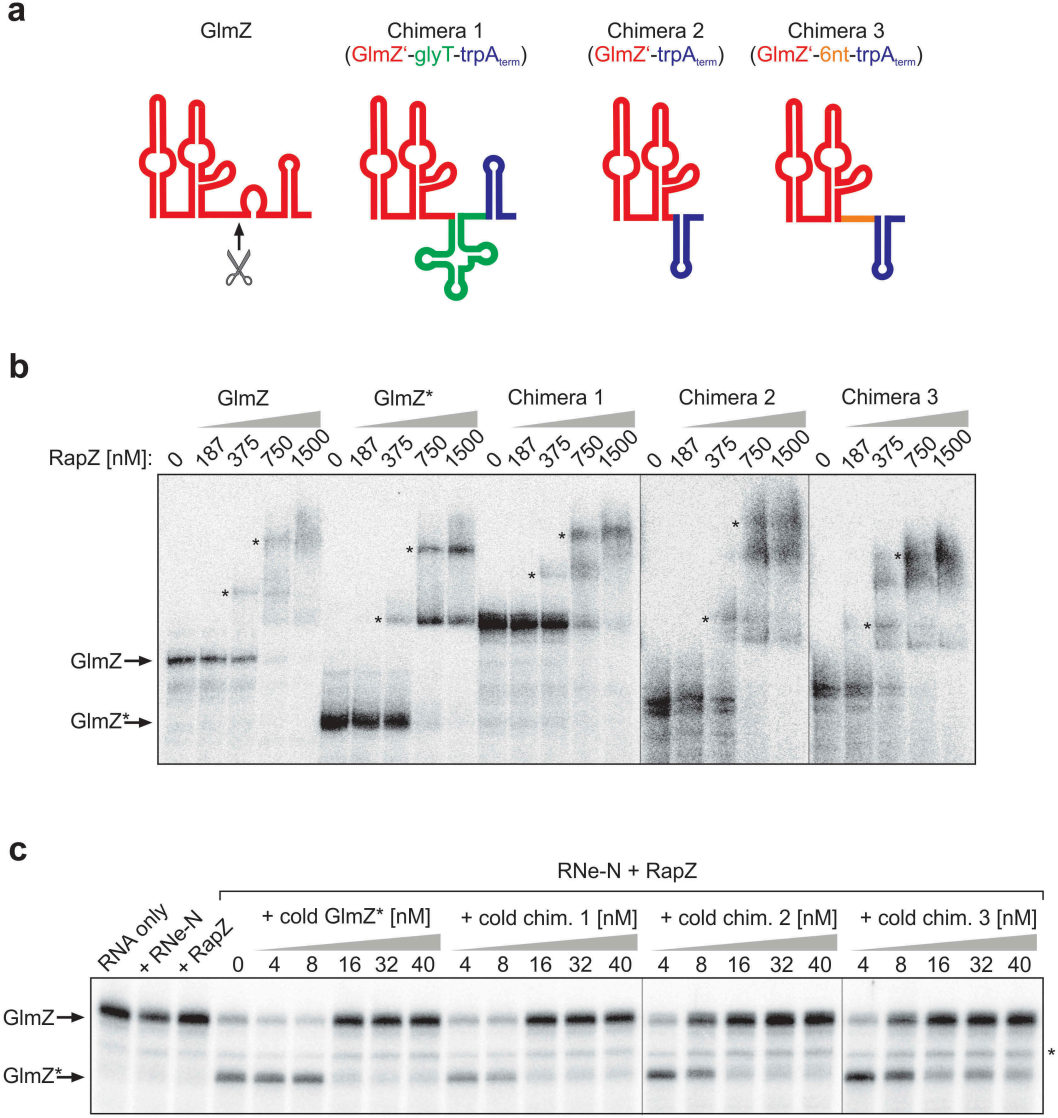
sRNA chimeras mimicking GlmZ* bind RapZ efficiently and counteract cleavage of full-length GlmZ by RNase E *in vitro*. (a) Schematic representation of the sRNA chimeras used to mimic GlmZ*. Parts derived from GlmZ are depicted in red, the *trpA* terminator is shown in blue, the *glyT* tRNA in green and the 6 nt BglII site insertion in chimera 3 is in orange. The structure of full-length GlmZ including location of the processing site (marked with an arrow) is shown for comparison. (b) EMSA demonstrating that RapZ binds GlmZ, GlmZ* and the sRNA chimeras with comparable affinities. α-^32^P-UTP labelled full-length GlmZ, GlmZ* and sRNA chimeras were incubated with incremental concentrations of RapZ, respectively, and binding reactions were subsequently separated on non-denaturing PAA gels and analysed by phospho-imaging. In case of chimeras 2 and 3, premature transcription termination additionally generated slightly shorter sRNA variants, which were similarly efficient bound as the full-length sRNAs and contributed to additional sRNA/RapZ complexes. It should be noted that complete binding of GlmZ required higher RapZ concentrations as previously reported [30,31], which may be attributed to the individual RapZ protein preparation used. (c) *In vitro* cleavage assay demonstrating that the various sRNA chimeras impede processing of full-length GlmZ by RapZ/RNase E similarly efficient as GlmZ*. Same assay as in Figure 2B, but as a difference the various non-labelled sRNA chimeras were added.

We wanted to complement the *in vitro* observations with evidence that accumulation of GlmZ* indeed limits ongoing processing of full-length GlmZ *in vivo*. As termination is required, it is impossible to generate primary transcripts *in vivo* corresponding to GlmZ*. Another possibility to increase abundance of GlmZ* is to block its degradation. GlmZ half-life measurements following rifampicin treatment indicated that GlmZ* is more stable as compared to full-length GlmZ (Figure 2(c) left). Quantification revealed t_1/2_ values of 2.2 ± 0.2 min for full-length GlmZ and 6.5 ± 2.7 min for GlmZ* in the *wild type* strain (Figure 2(c) right). The long half-life explains why GlmZ* remains detectable under steady state conditions and accumulates when processing is stimulated through elevated RapZ levels (see Figure 1). Consequently, GlmZ* exists long enough allowing it to compete with full-length GlmZ for interaction with RapZ. Unfortunately, our search for RNases responsible for turning over GlmZ* was unsuccessful. Absence of the major RNases PNPase, RNase III, RNase G, RNase R or RNase II did not cause accumulation of GlmZ*, respectively (Figure S1). Thus, these RNases have apparently no role for turnover of GlmZ* or redundant enzymes compensate. Therefore, we sought to mimic GlmZ* by using chimeras consisting of a heterologous transcriptional terminator fused to the 3ʹ end of GlmZ*. Such chimeras should fulfil two criteria: First, they must possess sizes allowing for their discrimination from endogenously produced full-length GlmZ and GlmZ* in Northern analyses. Second, they should not themselves be cleaved by RapZ/RNase E as this would liberate the GlmZ portion that is indistinguishable from GlmZ* derived from endogenously encoded GlmZ. We have previously shown that RNase E cleaves GlmZ at a fixed position in the single stranded region (i.e. 6 or 7 nucleotides) downstream of the second stem loop regardless of sequence composition at the cleavage site (Figure 3(a) left [31]). As sequence variation has no impact, we reasoned to prevent cleavage by reducing or removing the single-stranded region following the second stem loop. Lastly, we constructed three different chimeras, all consisting of the two first stem loops of GlmZ at the 5ʹ end and the transcriptional terminator of the *trpA* gene at the 3ʹ end, but with different sequences between these modules to also account for a possible steric hindrance of RapZ binding by the adjacent terminator (Figure 3(a)). Chimera 1 carries the highly structured *glyT* tRNA inserted between the sequences corresponding to GlmZ* (nt 1–155) and the *trpA* terminator, respectively (Figure 3(a)). In chimera 2, the *trpA* terminator was fused directly to the 3ʹ end of the central stem loop of GlmZ. Chimera 3 is similar, but additionally carries 6 foreign nucleotides between the central stem loop of GlmZ and the *trpA* terminator (Figure 3(a)).

### GlmZ chimeras mimicking GlmZ* inhibit processing of full-length GlmZ *in vitro* and *in vivo* through sequestration of RapZ

First, we tested whether the various sRNA chimeras are efficiently bound by RapZ and able to counteract processing of GlmZ by RapZ/RNase E *in vitro*. EMSA revealed no major differences in the binding properties between full-length GlmZ, GlmZ* and the chimeras: All these RNAs are bound by RapZ with comparable affinities (Figure 3(b)). Depending on the RapZ concentration multiple higher weight complexes became detectable that may refer to RapZ oligomers binding different numbers of GlmZ molecules. We recently showed that active RapZ is a tetramer possessing four RNA-binding surfaces [29]. When tested in the cleavage assay *in vitro*, the various chimeras inhibited processing of GlmZ by RapZ/RNase E similarly efficient as GlmZ*. In all cases, at least 16 nM of the respective competitor sRNA were required to completely block cleavage of GlmZ in an assay containing 25 nM RNase E-NTD and 20 nM RapZ (Figure 3(c)).

**Figure 4.**
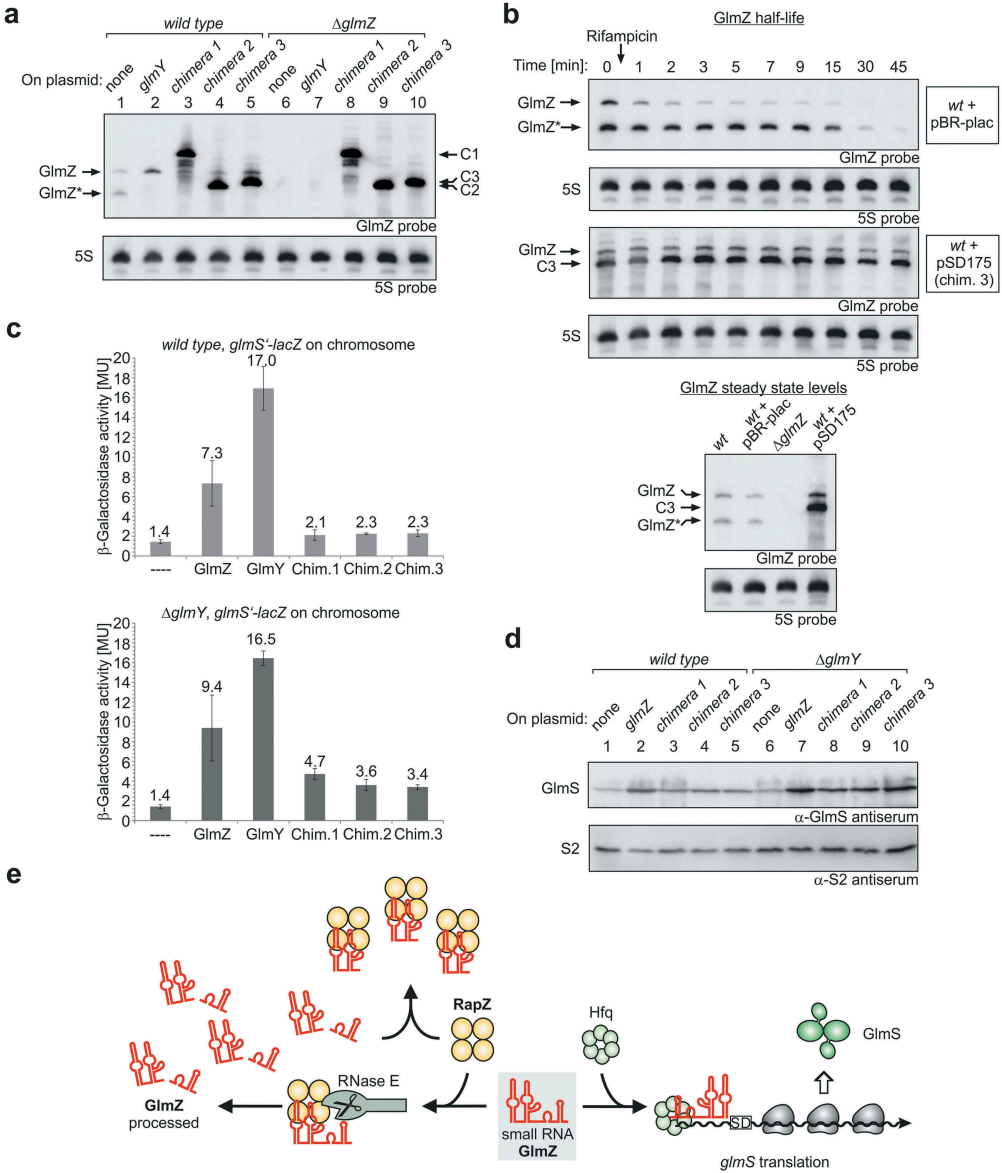
The sRNA chimeras mimicking GlmZ* counteract GlmZ processing *in vivo* thereby augmenting GlmS levels. (a) Plasmid-driven expression of the sRNA chimeras inhibits processing of endogenously encoded GlmZ. Strains R1279 (*wild type*, lanes 1–5) and strain Z45 (*ΔglmZ*, lanes 6–10) harbouring plasmids encoding GlmY (pYG83, lanes 2 and 7), sRNA chimera 1 (pSD164, lanes 3 and 8), chimera 2 (pSD174, lanes 4 and 9), chimera 3 (pSD175, lanes 5 and 10) or the empty plasmid pBR-plac (lanes 1 and 6) were grown in LB to exponential phase for isolation of total RNA. 4 μg total RNA each were analysed by Northern blotting using probes directed against GlmZ (top panel) and 5S rRNA (bottom panel). (b) sRNA chimera 3 counteracts degradation of full-length GlmZ. Northern blot addressing decay of full-length GlmZ in *wild type* strain R1279 in the presence of the empty plasmid pBR-plac (top panel) or plasmid pSD175 encoding chimera 3 (third panel from top). The transformants were grown to exponential phase and treated with rifampicin to stop transcription. Samples were removed at indicated times for isolation of total RNA, which was analysed by Northern blotting using probes directed against GlmZ and the 5S rRNA. To verify that the slower migrating band detected for transformant R1279/pSD175 corresponds to full-length GlmZ, the total RNA sample obtained before rifampicin addition (time 0) was analysed by Northern blotting (bottom panel) alongside total RNA samples of *wild type* strain R1279 (lane 1), R1279 carrying the empty plasmid pBR-plac (lane 2) and of the *ΔglmZ* mutant strain Z45 (lane 3). (c) Expression of the sRNA chimeras increases expression of a *glmS’-lacZ* reporter fusion, which becomes more pronounced in a *ΔglmY* mutant strain. Strains Z8 (*wild type*) and Z884 (*ΔglmY*) were employed, which carry a *glmS’-lacZ* fusion integrated into the *λattB* site on the chromosome. The strains harboured the plasmids described in (a) as indicated. Additionally, transformants carrying plasmid pYG84 overexpressing GlmZ were included for comparison. (d) Effect of the sRNA chimeras on GlmS protein levels. Protein extracts were prepared from the *wild type* strain R1279 and the *ΔglmY* mutant strain Z95, which harboured plasmid pBR-plac (empty plasmid, lanes 1 and 6), pYG84 (GlmZ, lanes 2 and 7), pSD164 (chimera 1, lanes 3 and 8), pSD174 (chimera 2, lanes 4 and 9) or pSD175 (chimera 3, lanes 5 and 10). The extracts were subjected to Western blotting using antisera against GlmS (top panel) and S2 protein (bottom panel). (e) Model illustrating feedback regulation of GlmZ processing by processed GlmZ. Tetrameric RapZ (yellow) recruits RNase E (grey) to elicit cleavage of bound full-length GlmZ. Processed GlmZ retains the elements required for interaction with RapZ and sequesters RapZ when accumulating to high levels. This reduces ongoing processing of GlmZ, which is then free to associate with Hfq (green hexamer) and to stimulate *glmS* expression by base-pairing. This mechanism may absorb fluctuations in RapZ and RNase E availabilities thereby setting a robust basal *glmS* expression level.

To test whether the various chimeras counteract processing of GlmZ *in vivo*, the fusion RNAs were expressed from plasmid pBR-plac under control of the *P_LlacO-1_* promoter. *Wild type* and *ΔglmZ* strains harbouring these plasmids were grown to the exponential growth phase and total RNAs were isolated and subjected to Northern analysis using a probe capable of detecting both endogenous GlmZ as well as the various chimeras. Assessing the RNAs in the *ΔglmZ* strain showed that all chimeras migrated in the gel at positions different from full-length and processed GlmZ allowing for their discrimination (Figure 4(a), compare lanes 8–10 with lane 1). Moreover, no processing products in the size of GlmZ* became detectable, confirming that RNase E is unable to cleave the chimeras downstream of the central stem loop. Importantly, in the *wild type* strain, expression of the various chimeras caused disappearance of the processed form of endogenous GlmZ and simultaneously led to a slight increase in abundance of full-length GlmZ (Figure 4(a), compare lanes 3–5 with lane 1). Analysis of these samples using a probe specific for the 3ʹ end of GlmZ corroborated increased abundance of full-length GlmZ upon expression of the sRNA chimeras (Figure S2, compare lanes 3–5 with lane 1).

To determine whether the latter increase in abundance results from slower GlmZ degradation, GlmZ half-life in the strain expressing chimera 3 was determined (Figure 4(b)). Notably, presence of chimera 3 led to a pronounced increase of full-length GlmZ half-life as compared to the strain carrying the empty plasmid (Figure 4(b), compare panels 1 and 3 from top). To corroborate once again that the slower migrating band corresponds to full-length GlmZ, the total RNA (isolated at *t* = 0) was separated alongside total RNAs isolated from the *wild type* strain containing no plasmid or the empty vector (Figure 4(b) bottom). Comparison of signal intensities confirmed up-regulation of full-length GlmZ in the strain producing chimera 3 once again. Taken together, our results indicate that the sRNA chimeras mimicking GlmZ* counteract RapZ-mediated cleavage of full-length GlmZ *in vitro* and *in vivo*, thereby increasing GlmZ steady state levels.

### Expression of the sRNA chimeras mimicking Glmz* augments Glms synthesis

To investigate the impact of the sRNA chimeras on *glmS* expression, we first assessed GlmS protein levels in the *wild type* strain. Western blotting analysis of total protein extracts revealed somewhat increased GlmS levels in cells expressing the sRNA chimeras as compared to the empty plasmid control (Figure 4(d), compare lane 1 with lanes 3–5). Quantification of signal intensities and their normalization to S2 protein levels indicates a two- to four-fold increase relative to the control (Figure S3). However, upregulation of GlmS levels by the sRNA chimeras was less pronounced as compared to cells overproducing GlmZ, which can directly activate *glmS* translation through base-pairing (Figure 4(d), compare lanes 2 with 3–5; Figure S3). To confirm the effects of the sRNA chimeras on *glmS* expression, we used strains carrying an ectopic *glmS’-lacZ* reporter fusion in the chromosome. In addition, these strains also harboured the plasmids encoding the various GlmZ chimeras or GlmZ or GlmY for comparison, respectively. Samples were harvested for determination of β-galactosidase activities as well as for extraction of RNA. Northern blotting of total RNAs proved once again that the sRNA chimeras counteract processing of endogenous GlmZ (Figure S4). Plasmid-driven expression of GlmY upregulated *glmS’-lacZ* expression 12-fold as compared to the strain carrying the empty plasmid, whereas overexpression of GlmZ increased enzyme activities 5.2-fold (Figure 4(c) top, columns 1–3), which is in agreement with previous results [31]. Expression of the various sRNA chimeras led to a minor, that is ~1.5-fold upregulation of *glmS* expression (Figure 4(c) top, compare columns 4–6 with 1).

However, it is reasonable to assume that the endogenous sRNA GlmY, whose levels are determined by the intracellular GlcN6P concentration [32,33], may compensate in the latter case for most of the effects generated by the sRNA chimeras. That is, upregulation of GlmS by the sRNA chimeras may increase the GlcN6P concentration, which will in turn decrease the level of decoy sRNA GlmY, thereby redirecting RapZ to recruit more GlmZ molecules to processing. To learn whether the latter feedback mechanism that essentially provides GlcN6P homeostasis indeed masks the regulatory effects caused by the sRNA chimeras, we expressed the sRNA chimeras in a *ΔglmY* mutant. Indeed, in this case, presence of the various sRNA chimeras had a stronger impact, increasing *glmS’-lacZ* expression two- to three-fold and leading to four- to five-fold higher GlmS protein levels (Figure 4(c) bottom diagram; Figure 4(d), lanes 6–10; Figure S3).

Taken together, our data show that accumulation of an RNA corresponding to the processed form of GlmZ limits further processing of full-length GlmZ via sequestration of RapZ (Figure 4(e)). Thus, a sRNA cleavage product feedback regulates its own production via processing, which is a novel activity for a processed sRNA in bacteria. This feedback regulation may serve to buffer noise in RapZ and RNase E availabilities, thereby ensuring that a low basal level of the essential enzyme GlmS is always maintained (Figure 4(e)). This mechanism may be in particular important when GlmY levels are low, e.g. when the QseE/QseG/QseF three-component system, which controls *glmY* transcription, is in the OFF state [34,35]. Under these conditions, accumulation of GlmZ* may limit further processing of full-length GlmZ thereby maintaining a robust GlmS basal level. Interestingly, a similar regulatory scenario has also been proposed for human microRNAs [36]. In this case, miRNAs such as miR-21–5p were shown to inhibit their own biogenesis from the corresponding miRNA precursors, at least *in vitro*. Mechanistically, it is speculated that this regulation involves binding of the mature miRNA to Dicer, similar to the mechanism described here, which relies on binding of GlmZ* to RapZ. Thus, autoregulatory loops in which processed RNAs control their own production by binding to the processing enzymes could represent a widespread principle operating in all domains of life.

## Materials and methods

### Strains, plasmids and growth conditions

*E. coli* strains were routinely grown in Lysogeny broth (LB medium) at 37°C under agitation (165 rpm). When required, antibiotics were added at following concentrations: ampicillin (100 μg/ml), kanamycin (30 μg/ml), spectinomycin (50 µg/ml) and chloramphenicol (15 μg/ml). Expression of genes controlled by *P_Ara_* promoter was induced by 0.2% arabinose and of genes controlled by *P_tac_* or *P_LlacO-1_* promoter with 1 mM IPTG. *E. coli* strains and plasmids used in this study are described in Table 1 and oligonucleotides are listed in Table S1 under ‘Supplemental Material’. Strain Z946 was constructed by transducing the *pnp::Tn5* allele of strain JC357 into strain Z854 using phage T4GT7 [37]. Construction of recombinant plasmids is described under ‘Supplemental Material’.

**Table 1.** Strains and plasmids used in this study.

Name	Genotype or relevant structure^a^	Reference
Strains:		
BW25113	Δ(*araD-araB*)567, Δ*lacZ*4787(::*rrnB*-3), *λ^−^,rph-1*, Δ(*rhaD-rhaB*)568, *hsdR*514	[39]
IBPC633	as N3433, but *rnc*105, *nadB*51::Tn*10* (*tet*)	[40]
IBPC935	as N3433, but *rng::cat*	[41]
JC357	F^−^ *argG6, metB1, his1, leu6, mtl2, xyl7, malA1, gal6, lacY1, tonA2, tsx1*, λ^R^, λ^−^, *supE44, rpsL, recA, pnp*::Tn5 (kan^R^)	[42]
JW1279	as BW25113, but *Δrnb-723::kan*	[39]
JW5741	as BW25113, but *Δrnr-729::kan*	[39]
N3433	HfrH, *lacZ43, λ^−^, relA1, spoT1, thi1*	[43]
R1279	CSH50 *Δ(pho-bgl)201, Δ(lac-pro), ara, thi*	[44]
XL1-blue	*recA1, endA1, gyrA96, thi-1, hsdR17, relA1, supE44, lac, F’*[*proAB lacI^q^ lacZΔM15 Tn10*]	Laboratory stock
Z8	as R1279, but *attB::[aadA, glmS*-5′::*lacZ*], *strp^R^, F‘*(*lacI^q^*)	[28]
Z37	as R1279, but *ΔrapZ*	[28]
Z39	as R1279, but *ΔglmZ, attB:*:[*aadA, glmS*-5′::*lacZ*], *strp^R^, F‘*(*lacI^q^*)	[31]
Z45	as R1279, but *ΔglmZ*	[28]
Z95	as R1279, but *ΔglmY::cat*	[32]
Z106	as R1279, but *ΔglmZ, ΔglmY*	[30]
Z854	MG1655 *rph^+^, ilvG^+^, ΔlacZ, λattB*::[*aadA* (spec^R^), *glmS −5*‘::*lacZ*]	[33]
Z884	as R1279, but *ΔglmY, attB:*:[*aadA, glmS*-5′::*lacZ*], *strp^R^, F‘*(*lacI^q^*)	[31]
Z946	as Z854, but *pnp*::Tn5 (kan^R^)	T4GT7 (JC357) → Z854; this work
Plasmids:		
pACA-RNA43^SD^	3´-terminal 54 nts of *rrnB* followed by *glyT* and the *trpA* terminator in pBAD33	[45]
pBAD33	*P_Ara_*, MCS 2, *cat*, ori p15A	[46]
pBGG61	*rapZ* (−17 to +855) under *P_Ara_* control in pBAD33	[30]
pBGG164	*strep-rapZ* under *P_tac_* control, *lacI^q^, bla, ori* ColEI	[47]
pBGG190	*His_10_-ptsN* under *P_tac_* control, *lacI^q^, bla, ori* ColEI	[48]
pBGG237	*Strep-tag* under *P_tac_* control, *lacI^q^, bla, ori* ColEI	[47]
pBR-plac	IPTG inducible artificial *P_LlacO-1_* promoter in pBR322; allows to start sRNA transcription at authentic +1 position	[49]
pRne529-N	*His_6_-rne* (+1 to +1587) in pET16b, *bla*, ori ColEI	[50]
pSD23	*His_10_-rne* (+1 to +1587) under *P_tac_* control, *lacI^q^, bla*, ori ColEI	this work
pSD164	*glmZ* (1–155)-*glyT-trpA*_term_ fusion in pBR-plac	this work
pSD174	*glmZ* (1–146)-*trpA*_term_ fusion in pBR-plac	this work
pSD175	*glmZ* (1–146)-BglII*-trpA_term_* fusion in pBR-plac	this work
pYG83	*glmY* in pBR-plac	[31]
pYG84	*glmZ* in pBR-plac	[31]
pYG135	*strep-rne* (+1 to +1587) under *P_tac_* control, *lacI^q^, bla, ori* ColEI	this work

^a^ORI: origin of replication; MCS: multiple cloning site

### RNA extraction, Northern blotting and sRNA half-life determination

Total RNA was extracted from exponentially growing cells using the ReliaPrep RNA Cell Miniprep System (Promega). For determination of GlmZ half-life, 500 μg/ml rifampicin was added to the culture. A sample harvested at the time of rifampicin addition corresponded to *t* = 0 min. Additional samples following rifampicin addition were collected for RNA extraction at the times indicated in Figure 2(c). Isolated total RNA was mixed with 2× RNA loading dye (95% formamide, 0.5 mM EDTA, 0.025% SDS, 0.025% bromophenol blue, 0.025% xylene cyanol) and separated by gel electrophoresis under denaturing conditions (7M urea, 6% acrylamide, 1× TBE) in 0.5× TBE as running buffer. Afterwards, RNA was transferred to a positively charged nylon membrane (Hybond H^+,^ GE Healthcare) via electroblotting in 0.5× TBE for 1 h at 120 mA and crosslinked by exposure to 254 nm UV radiation. Digoxigenin (DIG)-labelled RNA probes were generated by *in vitro* transcription using T7 RNA polymerase (NEB) and DIG RNA labelling mix (Roche Diagnostics). Templates for probe synthesis were generated by PCR using primers BG230/BG231 for *glmZ*, BG1795/BG1796 for the *glmZ* 3ʹ-end (nt 153–202) and BG287/BG288 for *rrfD*. Signal detection was achieved using an anti-DIG AP-conjugated antibody and CDP* as substrate, following the manufacturer´s instructions (Roche Diagnostics). Signals were quantified using software ImageQuant TL 8.1 (GE Healthcare).

### SDS-PAGE and Western blotting

Total protein extracts were prepared in SDS sample buffer and samples corresponding to 0.0625 OD_600_ units of the cultures were separated on 12.5% SDS-polyacrylamide gels, respectively. Subsequently, proteins were transferred to a PVDF membrane (Amersham) via semidry blotting (Peqlab, 90 min at 120 mA). Following blotting, the SDS-PAA gels were stained with Coomassie Brilliant Blue R-250. The PVDF membranes were incubated with the required primary antisera (S2 antiserum diluted 1:5000; GlmS antiserum diluted 1:10,000 [38]; RapZ antiserum diluted 1:3000 [30]; anti-Strep antiserum diluted 1:20,000 (Promokine)) at 4°C overnight. The primary antibodies were detected by using rabbit IgG-AP conjugated secondary antibodies (Promega) diluted 1:100,000 and CDP* as substrate (Roche Diagnostics).

### Protein purification

Strep-RapZ and the His-tagged catalytic domain of RNase E (aa 1–529) were overproduced in strain Z106 lacking endogenous *glmY* and *glmZ* genes using plasmids pBGG164 and pSD23, respectively. The proteins were subsequently purified as described previously with minor modifications for Strep-RapZ [30,31]. Modifications include cell lysis by sonication, clearing of the lysate by centrifugation at 14,000 rpm (1 h, 4°C) and omission of dialysis. Purified proteins were mixed with glycerol (5% v/v final concentration), shock-frozen and stored at −80°C until use.

### *In vitro* transcription and labelling of small RNAs

Generation of ^32^P-UTP labelled sRNAs *in vitro* is described under ‘Supplemental Material’.

### EMSA

EMSAs were carried out as previously described [31]. Binding reactions were performed in 1× binding buffer (10 mM Tris-HCl pH 7.0, 100 mM KCl, 10 mM MgCl_2_) in a volume of 10 μl. The radiolabelled sRNA was mixed with 1 µg of yeast tRNA (Ambion), heat-denatured and briefly chilled on ice. Subsequently, appropriate RapZ protein dilutions (prepared in binding buffer) were added and samples were incubated for 30 min at 30°C. Thereafter, 2 µl of 5× native loading buffer (50% glycerol, 0.5 TBE, 0.2% bromophenol blue) were added and samples were separated on native polyacrylamide gels (6% PAA, 1× TBE) for 3 h at 4°C and 300 V using 0.5× TBE as running buffer. In case of competitive binding assays (Figure 2(a)), the radiolabelled sRNA and appropriate RapZ amounts were first co-incubated for 15 min at 30°C to allow for complex formation. Afterwards, non-radiolabelled competitor RNA was added in various concentrations and incubation was continued for 15 min at 30°C. Signals were detected by phospho-imaging (Typhoon FLA 9000, GE Healthcare).

### RNase E cleavage assay

RNase E *in vitro* cleavage assays were performed in 1× reaction buffer (25 mM Tris/HCl pH 7.5, 50 mM NaCl, 50 mM KCl, 10 mM MgCl_2_, 1 mM DTT) in a final volume of 10 μl as described earlier [31]. Briefly, radiolabelled GlmZ was mixed with 1 µg yeast tRNA (Ambion) and cold competitor RNA where appropriate. The RNA mix was heat-denatured, chilled on ice and incubated at 30°C for 5 min to allow for RNA folding. Afterwards, 20 nM RapZ was added and the samples were further incubated for 10 min at 30°C. Subsequently, 25 nM RNase E was added and incubation was continued for 30 min. The reactions were stopped by addition of 0.2 U proteinase K (NEB) and proteinase K buffer (100 mM Tris/HCl pH 7.5, 12.5 mM EDTA, 150 mM NaCl, 1% SDS) and incubation at 50°C for 30 min. Following the addition of 1 volume 2× RNA loading dye (95% formamide, 0.5 mM EDTA, 0.025% SDS, 0.025% bromophenol blue, 0.025% xylene cyanol) samples were separated on denaturing polyacrylamide gels (6% PAA, 7M urea, 1× TBE). Dried gels were analysed by phospho-imaging.

### Determination of β-galactosidase activity

β-Galactosidase activity assays were performed as previously described [51]. Shown values are the average of at least three measurements from independent cultures.
