# Supplementary material for: Feedback regulation of small RNA processing by the cleavage product
Source: RNA Biol. 2019 May 22;16(8):1055–65. doi: 10.1080/15476286.2019.1612693 (PMC6602413; doi:10.1080/15476286.2019.1612693)
Supplement: Supplemental Material [file krnb-16-08-1612693-s001.docx]

**SUPPLEMENTAL MATERIAL**

**for**

**Feedback regulation of small RNA processing by the cleavage product**

Svetlana Durica-Mitic^1^ and Boris Görke^1*^

^1^Department of Microbiology, Immunobiology and Genetics, Max F. Perutz Laboratories (MFPL), University of Vienna, Vienna Biocenter (VBC), Vienna, Austria

Short title: Feedback regulation of small RNA GlmZ processing

^*^Corresponding author

E-mail: boris.goerke@univie.ac.at

**Supplemental Figures**


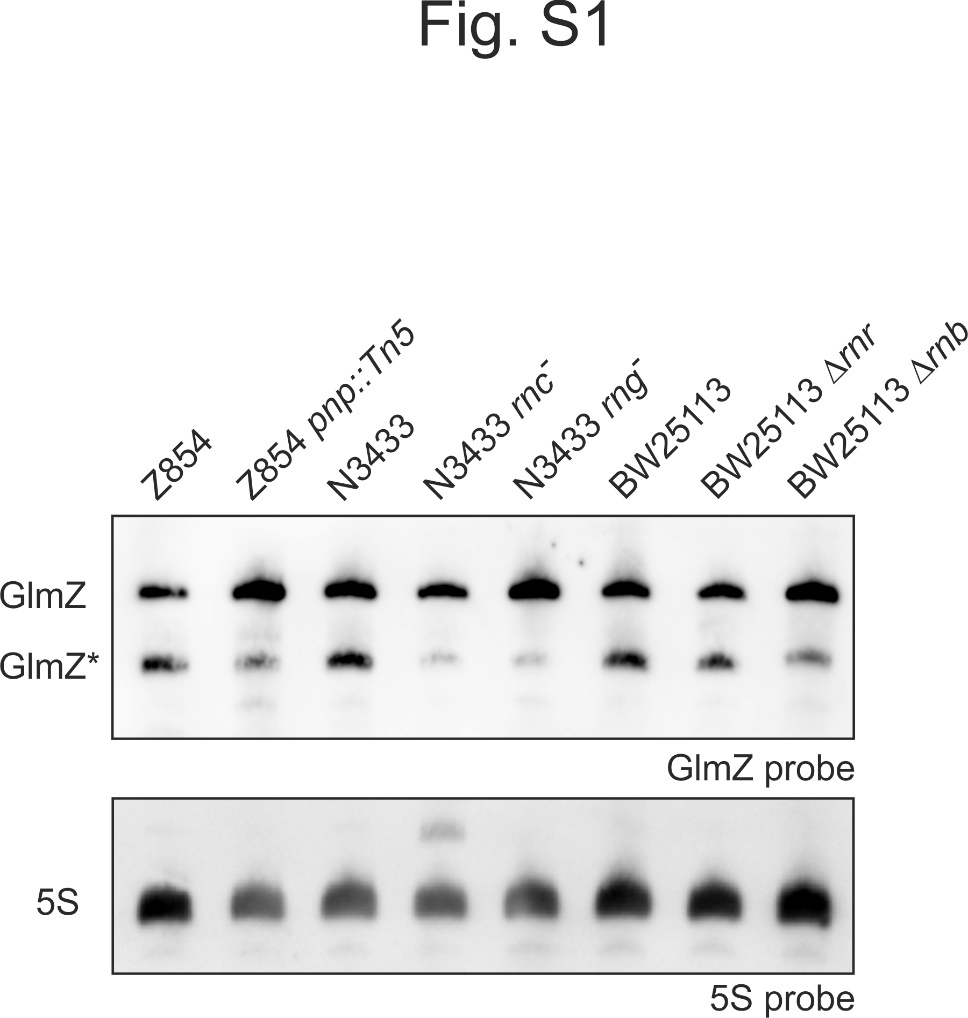


**Fig. S1.** The major RNases PNPase, RNase III, RNase G, RNase R and RNase II have no role for turnover of GlmZ*. Strains Z854 (lane 1), Z946 (lane 2), N3433 (lane 3), IBPC633 (lane 4), IBPC935 (lane 5), BW25113 (lane 6), JW5741 (lane 7) and JW1279 (lane 8) were grown in LB to exponential phase. Total RNAs were isolated and analyzed by Northern blotting using probes against GlmZ (top) and the 5S rRNA (bottom).


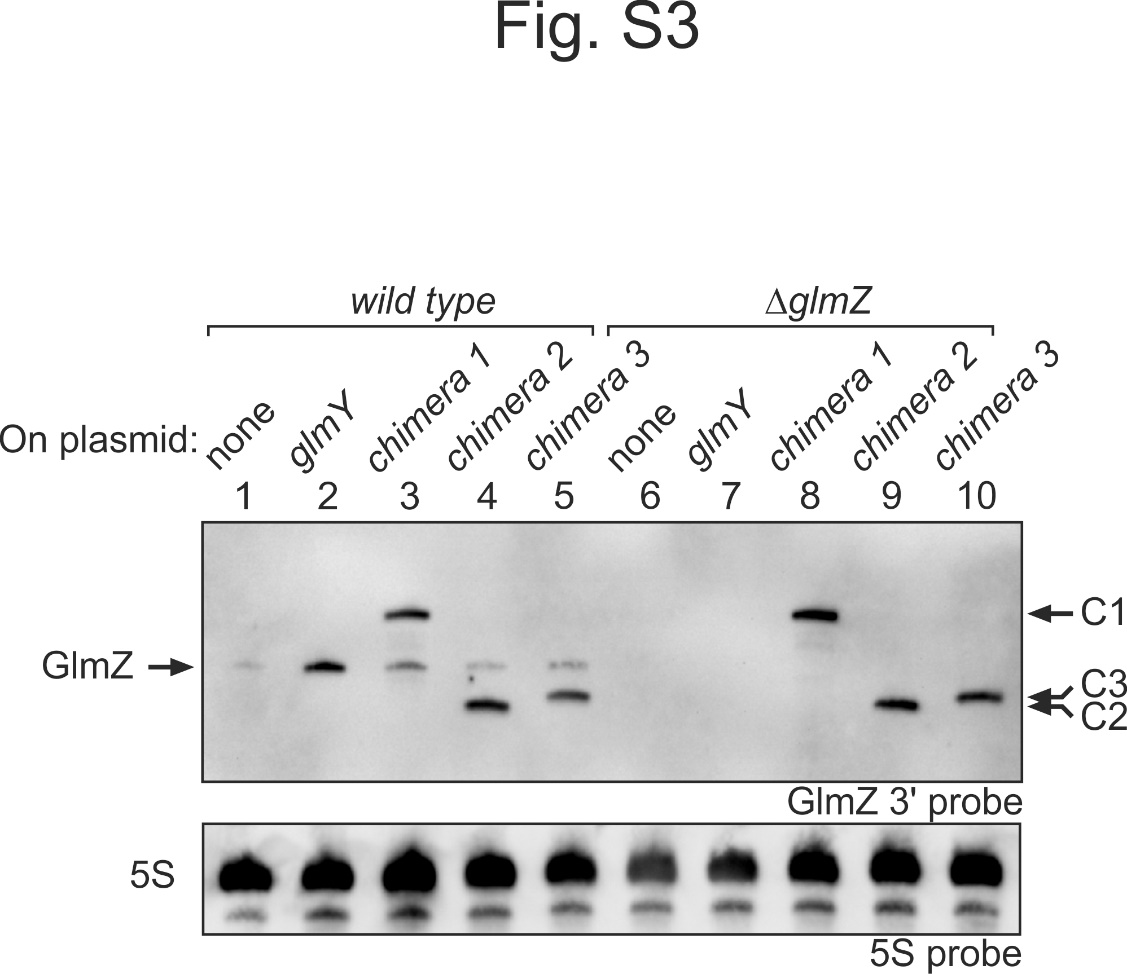


**Fig. S2.** Expression of the sRNA chimeras increases steady state levels of full-length GlmZ. The samples analyzed in Fig. 4A were reanalyzed using a probe directed against the 3’ end of GlmZ, which is not present in GlmZ*. Contrary to expectation, this probe preparation also detected the sRNA chimeras, albeit less efficiently as compared to the probe used in Fig. 4A. A likely explanation is that the probe preparation was contaminated with RNA probes covering the complete GlmZ sequence. As plasmid pYG84 carrying the full-length *glmZ* gene was used as template to generate the 74 nt long PCR fragment for *in vitro* transcription of the RNA probe, longer DNA strands complementary to the complete GlmZ were also produced through linear amplification by the reverse primer. The subsequent *in vitro* transcription might have further amplified these longer sequences on the RNA level, causing detection of the chimeric sRNAs.


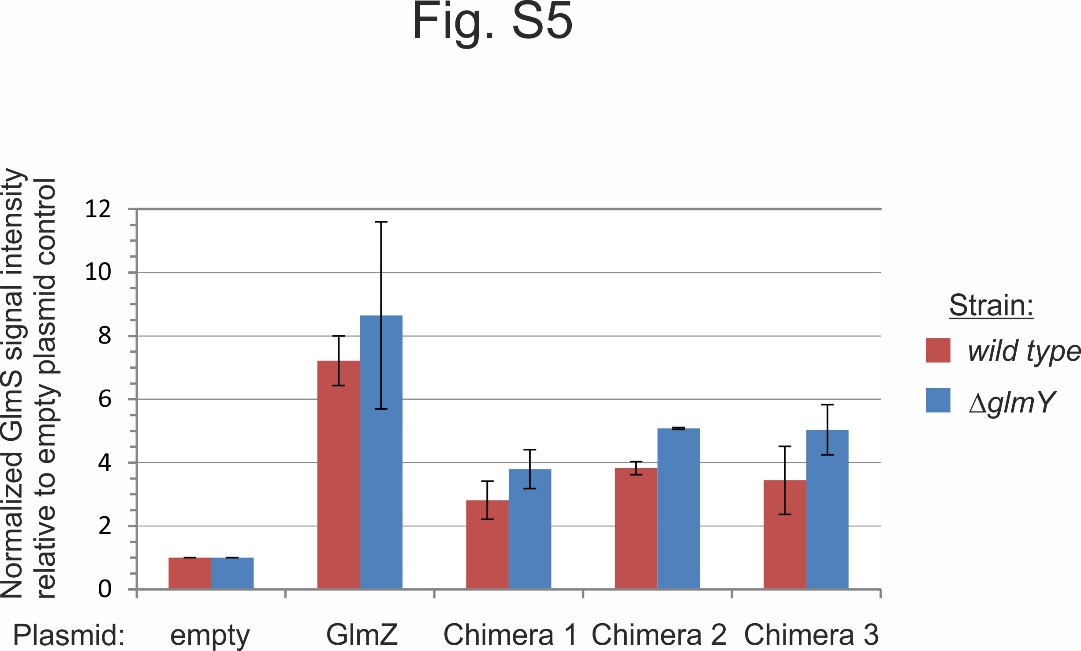


**Fig. S3.** Upregulation of GlmS protein levels by the sRNA chimeras. GlmS protein signal intensities in Fig. 4D and a biological replicate experiment were quantified and normalized to S2 signals. The graph shows the various GlmS signal intensities relative to the GlmS signal obtained for the strains carrying the empty plasmid pBR-pLac, which was set to 1. Quantification was performed using software ImageQuant TL 8.1 (GE Healthcare).


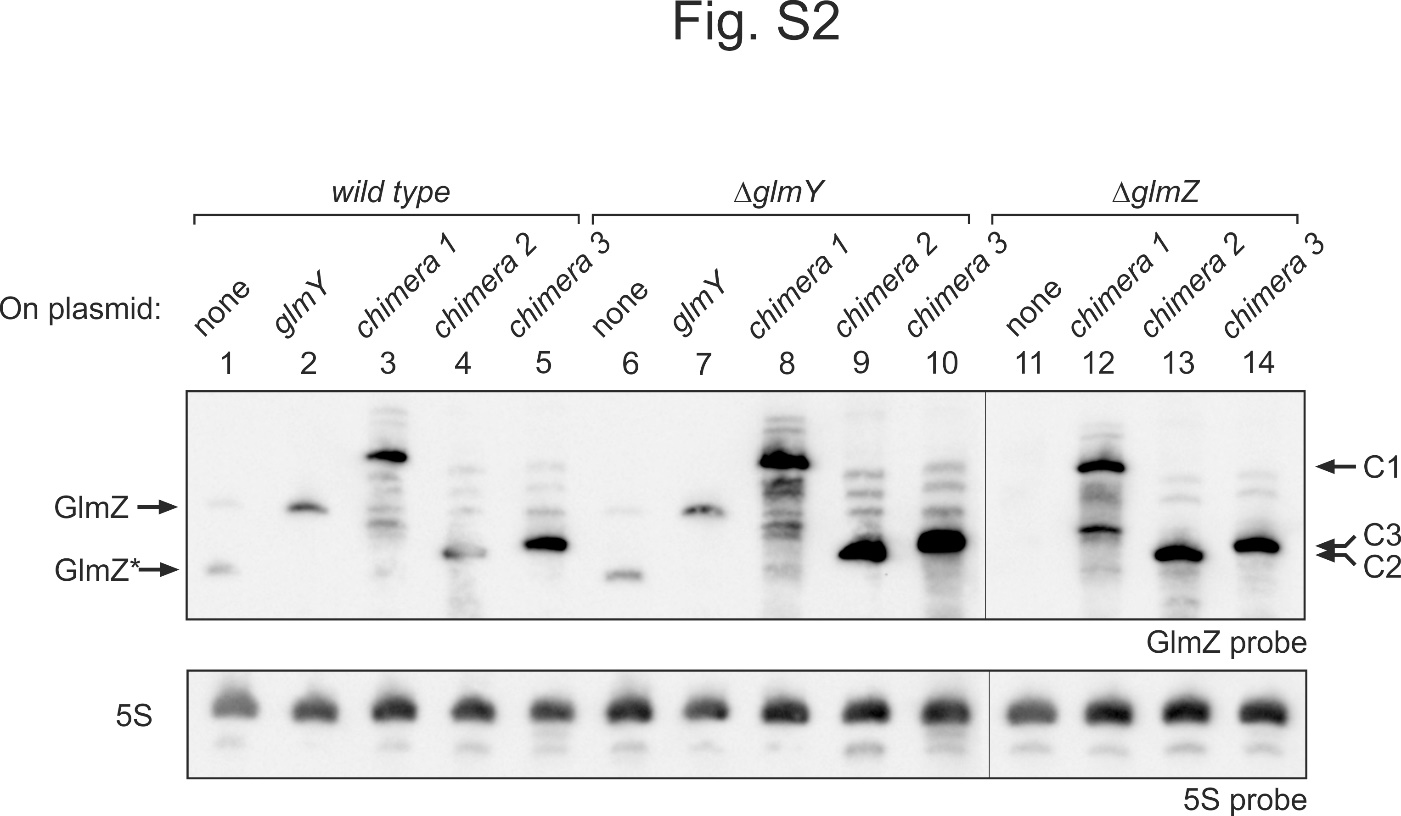


**Fig. S4.** Northern blot demonstrating inhibition of processing of endogenous GlmZ by the sRNA chimeras in the bacteria analyzed in Fig. 4C. In addition, strain Z39 (*ΔglmZ*, lanes 11-14) carrying the plasmids encoding the sRNA chimeras was analyzed to demonstrate accumulation of endogenously encoded full-length GlmZ in the wild-type and *ΔglmY* strains. The blot was analyzed using probes against GlmZ (top) and the 5S rRNA (bottom).

**Supplemental Table**

**Table S1**. Oligonucleotides used in this study.

| Primer | Sequence^a^ | Res. Sites | | Position^b^ |
| --- | --- | --- | --- | --- |
| BG230 | GTAGATGCTCATTCCATCTC | |  | *glmZ* 1-20 |
| BG231 | CTAATACGACTCACTATAGGGagAAAACAGGTCTGTATGACAAC | |  | *glmZ* 172-152 |
| BG287 | TGCCTGGCGGCCGTAG | |  | *rrfD* 1-16 |
| BG288 | CTAATACGACTCACTATAGGGagagcctggcagttccctac | |  | *rrfD* 118-102 |
| BG444 | CTAATACGACTCACTATAGGGagAgtagatgctcattccatctcttatg | |  | *glmZ* 1-25 |
| BG445 | AAAAAAACGCCTGCTCTTATTACGGAGC | |  | *glmZ* 207-180 |
| BG446 | CTAATACGACTCACTATAGGGAGAAGTGGCTCATTCACCGACTTATGTC | |  | *glmY* 1-25 |
| BG448 | AAGGCGGTGCCTAACTCGACG | |  | *glmY* 148-127 |
| BG471 | CAACAAGTGGGTGCTTCACTC | |  | *glmZ* 155-135 |
| BG1102 | GCGGGACGTCGTAGATGCTCATTCCATCTCTTATG | | AatII | *glmZ* 1-25 |
| BG1161 | ggctgctagcatgaaaagaatgttaatcaacgc | | NheI | *rne* 1-23 |
| BG1162 | GGCTtctagattacagcgcaggttgttccgg | | XbaI | *rne* 1587-1570 |
| BG1676 | GAGTGAAGCACCCACTTGTTGgcgggcatcgtataatggctattac | |  | *glmZ* 135-155, *glyT* 1-25 |
| BG1677 | ggcgaattcaagcttaaaaaaaagcccgctcattag | | EcoRI, HindIII | *trpA_term_* 28-8 |
| BG1721 | ggcgaattcaagcttaaaaaaaagcccgctcattagGCGGGCTGGTGCTTCACTCAACGTTGTG | | EcoRI, HindIII | *trpA_term_*, *glmZ* 146-126 |
| BG1722 | ggcgaattcaagcttaaaaaaaagcccgctcattagGCGGGCTAGATCTGGTGCTTCACTCAACGTTGTG | | EcoRI, HindIII, BglII | *trpA_term_*, BglII, *glmZ* 146-126 |
| BG1795 | TTGTCATACAGACCTGTTTTAACG | |  | *glmZ* 153-176 |
| BG1796 | CTAATACGACTCACTATAGGGAGAaacgcctgctcttattacggag | |  | *glmZ* 202-181 |

^a^Restriction sites are underlined. The recognition site for T7 RNA polymerase is underlined by a dashed line. ^b^Positions are relative to the first nucleotide of the respective gene.

**Supplemental Materials and Methods**

**Construction of plasmids**

*E. coli* strain Xl1-blue was used for cloning procedures. Plasmids pSD164, pSD174 and pSD175 are derivatives of plasmid pBR-plac encoding the various sRNA chimeras. DNA fragments carrying the sequences of sRNA chimera 2 and 3 were obtained by PCR using forward primer BG1102 and the reverse primers BG1721 and BG1722, respectively, and the *glmZ* gene as template. Reverse primer BG1721 added the *trpA* terminator sequence to the 3’ end of the sequence encoding the first two stem loops of GlmZ (nt 1-146), whereas reverse primer BG1722 additionally introduced a BglII site between the GlmZ (1-146) and *trpA* terminator sequences. The PCR fragments were inserted between the AatII/EcoRI sites on plasmid pBR-plac resulting in plasmids pSD174 and pSD175, respectively. The DNA fragment carrying the sequence of sRNA chimera 1 was generated by overlap extension PCR. A DNA fragment corresponding to nt 1-155 of the *glmZ* gene was amplified by PCR using the primer pair BG1102/BG471. In addition, a DNA fragment comprising a fusion of *glyT* to the *trpA* terminator was generated via PCR using primers BG1676/BG1677 and plasmid pACA-RNA43^SD^ as template. Primer BG1676 added 21 nt of the *glmZ* gene (nt 135-155) to the 5’ end of the *glyT-trpA_term_* fusion, thereby providing a sequence overlapping with the sequence of the aforementioned PCR fragment comprising *glmZ* 1-155. The two PCR fragments were purified, mixed and subjected to a further PCR using primers BG1102/BG1677. The resulting DNA fragment comprising the *glmZ*(1-155)-*glyT*-*trpA_term_* fusion gene was inserted between the AatII/EcoRI sites on plasmid pBR-plac resulting in plasmid pSD164. Construction of plasmid pSD23 overproducing the His_10_-tagged catalytic domain of RNase E (His_10_-Rne 1-529) involved two steps. First, the sequence encoding the catalytic domain of RNase E (*rne* nt 1–1587) was amplified by PCR using primers BG1161/BG1162 and plasmid pRne529-N as template. The PCR fragment was inserted between the NheI and XbaI sites on plasmid pBGG237 resulting in plasmid pYG135, which encodes Strep-Rne 1-529 under *P_tac_* promoter control. Next, the NheI-XbaI fragment of plasmid pYG135 comprising *rne* nt 1 – 1587 was used to replace the NheI-XbaI fragment (comprising gene *ptsN*) in plasmid pBGG190, resulting in plasmid pSD23. Plasmid constructions were confirmed by DNA sequencing.

***In vitro* transcription and labeling of small RNAs**

Radiolabeled and non-radiolabeled sRNAs were generated by *in vitro* transcription of appropriate PCR fragments using T7 RNA polymerase. The PCR templates for *in vitro* transcription were obtained using primers BG444/BG445 (full-length GlmZ), BG444/BG471 (GlmZ*), BG446/BG448 (processed GlmY) and BG444/BG1677 (chimeras 1, 2 and 3). For sRNA labeling, 1 μg of the respective PCR fragment was used in a 20 μl *in vitro* transcription reaction containing 50 units T7 RNA polymerase, 40 units RiboLock RNase inhibitor (Thermo Fisher Scientific), 0.5 mM ATP, 0.5 mM CTP, 0.5 mM GTP, 0.005 mM UTP and 20 μCi α-^32^P-UTP in 1×RNA Pol reaction buffer. The reaction was incubated at 37ºC for at least 2 h followed by removal of unincorporated nucleotides using Illustra Microspin G-50 columns (GE Healthcare). After addition of 2× RNA loading dye (95% formamide, 0.5 mM EDTA, 0.025% SDS, 0.025% bromophenol blue, 0.025% xylene cyanol), RNA was electrophoretically separated on a denaturing gel (7M urea, 6% acrylamide, 1× TBE) for 2 h at 300 V in 1× TBE as running buffer. Subsequently, the wet gel was subjected to phosphoimaging, thus enabling excision of the correct RNA product. Gel purification of RNA was achieved by addition of RNA elution buffer (20 mM Tris-HCl pH 7.5, 0.25 M sodium acetate, 1 mM EDTA, 0.25% w/v SDS), shock freezing on dry ice followed by overnight incubation at RT ([Nilsen, 2013](#_ENREF_1)). RNA was further extracted via phenol:CHCl_3_:isoamyl alcohol (25:24:1), precipitated with a mixture of ethanol:3M sodium acetate pH 5.2 (30:1) at -20°C for at least 2 h and dissolved in 20 μl RNase free water. Unlabeled sRNAs were produced using the same protocol, but with equal NTP concentrations (5 mM each NTP). Furthermore the nucleotide removal step was omitted and for gel purification, the denaturing gel was stained with ethidium bromide prior to excision of the sRNAs.

**Supplemental Reference**

Nilsen, T.W., (2013) Gel purification of RNA. *Cold Spring Harb. Protoc.* **2013**: 180-183.
